# Comparative analysis of medicinal plant *Isodon rubescens* and its common adulterants based on chloroplast genome sequencing

**DOI:** 10.3389/fpls.2022.1036277

**Published:** 2022-11-21

**Authors:** Zhongyu Zhou, Jing Wang, Tingting Pu, Jingjing Dong, Qin Guan, Jun Qian, Linchun Shi, Baozhong Duan

**Affiliations:** ^1^ College of Pharmaceutical Science, Dali University, Dali, China; ^2^ College of Life Science, Northeast Forestry University, Harbin, China; ^3^ Institute of Medicinal Plant Development, Chinese Academy of Medical Sciences, Peking Union Medical College, Beijing, China

**Keywords:** *Isodon rubescens*, chloroplast genome, species identification, molecular marker, phylogenetic

## Abstract

*Isodon rubescens* (Hemsley) H. Hara is the source of Donglingcao under the monograph *Rabdosiae Rubescentis Herba* in Chinese Pharmacopoeia. In the local marketplace, this medicine can be accidentally contaminated, deliberately substituted, or mixed with other related species. The contaminants of herbal products are a threat to consumer safety. Due to the scarcity of genetic information on *Isodon* plants, more molecular markers are needed to avoid misidentification. In the present study, the complete chloroplast (cp) genome of seven species of *Isodon* was sequenced, *de novo* assembled and characterized. The cp genomes of these species universally exhibited a conserved quadripartite structure, i.e., two inverted repeats (IRs) containing most of the ribosomal RNA genes and two unique regions (large single copy and small single copy). Moreover, the genome structure, codon usage, and repeat sequences were highly conserved and showed similarities among the seven species. Five highly variable regions (*trnS-GCU-trnT-CGU*, *atpH-atpI*, *trnE-UUC-trnT-GGU, ndhC-trnM-CAU*, and *rps15-ycf1*) might be potential molecular markers for identifying *I. rubescens* and its contaminants. These findings provide valuable information for further species identification, evolution, and phylogenetic research of *Isodon*.

## Introduction

*Isodon rubescens* (Hemsley) H. Hara belongs to the family Lamiaceae (Liu et al., 2017), which is listed in the Chinese Pharmacopoeia, and the Chinese name is “Donglingcao” (Chinese Pharmacopoeia Commission, 2020). The *Rabdosiae Rubescentis Herba* has crucial medicinal value in eliminating inflammation, reducing sore throats, and treating malignant tumors (Xue et al., 2020; Guan et al., 2021). The previous survey has revealed that *I. rubescens* is generally contaminated with common adulterants, such as *I. inflexus* (Thunb.) Kudô, *I. eriocalyx* (Dunn) Kudô, *I. excisus* (Maxim.) Kudô, *I. lophanthoides* (Buch.-Ham. ex D.Don) H.Hara, *I. coetsa* (Buch.-Ham. ex D.Don) Kudô, and *I. japonicus* (Burm.f.) H.Hara (Su et al., 2007; Xia et al., 2013; Ge et al., 2022). These adulterants are usually of poor quality and some might even be toxic (Xia et al., 2013; Duan et al., 2018). As the morphology of these species is similar, interchangeable, and indistinguishable, the identification of these species remains somewhat controversial, which may affect their safety and effectiveness in clinical use (Lazarski et al., 2014; Lin et al., 2019). Therefore, it is imperative to develop a method for accurately identifying *I. rubescens* and its common adulterants.

With the rapid development of molecular technology in recent years, molecular identification has made significant progress in Chinese medicine, especially molecular markers, which involve sequencing specific sections of the genome to identify differences between individuals of different species or populations (Roman and Houston, 2020). Recent studies have shown high levels of genetic variability within species of *Isodon* and an associated lack of phylogenetic resolution between different species (Zhong et al., 2010; Chen et al., 2021). Universal DNA markers, such as ITS, *psbA-trnH, trnD-trnT, rpl32-trnL*, and ETS have been used to identify *I. rubescens* and its related taxa (Xia et al., 2013; Yu et al., 2014; Chen et al., 2021). Moreover, according to Harris and Klooster (2011), they found that 11 microsatellite loci amplify reliably and are sufficiently variable for studying population genetics in *I. rubescens*. However, some common adulterants were not included in these studies. Therefore, more scientific and accurate identification methods must be developed. The chloroplast (cp) is an essential organelle that plays a crucial role in plant photosynthesis and biochemical processes (Bendich, 2004). Compared with the gene fragments, the cp genome is relatively conserved and slightly varied (Drouin et al., 2008; Ferrarini et al., 2013), which has been widely used for identifying *Paris*, *Polygonatum*, and its contaminants (Kawabe and Miyashita, 2003; Jiang et al., 2022; Wang et al., 2022). Recently, although the complete plastid genomes of *I. rubescens* has reported by Lian et al. (2022) and Yue et al. (2021), the focus of these papers was to compare the intraspecific variation or characterize one genome information. However, the use of cp genomes for comparing *Isodon* species with their common adulterants has not been reported.

Our study aims to: (i) contribute new fully-sequenced cp genomes in *Isodon* and improve the understanding of the overall structure of these genomes, (ii) perform comparative analyses and elucidate the phylogenetic evolution of *Isodon* cp genomes, and (iii) screen molecular markers to differentiate *I. rubescens* and its adulterants. In the current work, the complete cp genomes of seven *Isodon* species were sequenced, *de novo* assembled, and annotated. These genomes were then used in a comparative analysis of genome structure and evolution relationships. The data acquired in this study increase the genomic resources available for the *Isodon* genus and provide valuable information support for the phylogenetic analysis and identification of the *Isodon* genus, as well as safe medical applications of *I. rubescens*. This study is the first comprehensive research on identifying *I. rubescens* and its adulterants based on the cp genomes.

## Materials and methods

### Plant and DNA resources

The fresh, healthy leaves for seven species of *I. rubescens* and its common adulterants, including *I. inflexus*, *I. eriocalyx*, *I. excisus*, *I. lophanthoides*, *I. coetsa*, and *I. japonicus*, were collected from the Germplasm Resource Garden (Yunnan, China, 24°49′55″N, 102°48′58″E), Kunming Zhifen Biotechnology Co., Ltd. One individual sample of approximately 1.0 g of fresh leaves per plant species was gathered and stored in an ice-filled cooler or refrigerator (4°C) until DNA extractions could be performed. Professor Baozhong Duan authenticated the specimens, and the detailed sample information is available in **Supplementary Table S1** and **Figure S1**. The voucher specimens were deposited in the Dali University herbarium. Genomic DNA was extracted from tissue samples using the Plant Genomic DNA kit (Tiangen, Beijing, China) following the manufacturer’s protocol. The extracted DNA was quantified on high-sensitivity Qubit 4.0 fluorometry (Life Technologies, Inc.), and all PCR products were examined for the presence of amplified products in agarose gels.

### DNA sequencing, assembly, and annotation

For sequencing library preparation, we used thirty microlitres of high-quality (>100 ng/μL) DNA per individual. All libraries were sequenced on the Illumina NovaSeq system (Illumina, San Diego, CA). Paired-end sequence reads were trimmed to remove low-quality bases and adapter sequences in the Toolkit_v2.3.3 software. The cp genomes were assembled by GetOrganelle v.1.6.4, exploiting Bowtie2 v.2.4.4, SPAdes v.3.13.0, and Blast v.2.5.0 as dependencies (Jin et al., 2019). After assembly, two online annotation tools, CpGAVAS2 and GeSeq were used to annotate the circular cp genomes (Wick et al., 2015), and the annotated cp genome sequences were submitted to the GenBank database of the National Center for Biotechnology Information (NCBI) (**Table 1**). Gene maps of the cp genomes were produced with the online IRscope (https://irscope.shinyapps.io/Chloroplot/).

**Table 1:** Information of *Isodon* cp genome features.

Genome characterristics	Total length (bp)	GC content (%)	AT content (%)	LSC length (bp)	SSC length (bp)	IR length (bp)	GenBank accession
*I. inflexus*	152695	37.63	62.37	83558	17663	25722	OM808733
*I. eriocalyx*	152657	37.63	62.37	83546	17657	25727	OM808731
*I. excisus*	152643	37.64	62.36	83531	17656	25728	OM808732
*I. lophanthoides*	152208	37.61	62.39	83079	17729	25700	OM808735
*I. japonicus*	152238	37.60	62.40	83139	17699	25700	OM808734
*I. coetsa*	152441	37.63	62.37	83289	17670	25726	OM808730
*I. rubescens*	152690	37.62	62.38	83577	17661	25726	OM808736

### Repeat analysis

The GC content was analyzed using the Geneious 9.0.2 software (Kearse et al., 2012). Four kinds of the dispersed repeat sequence, including Forward (F), Reverse (R), Palindromic (P), and Complementary (C), were detected using the REPuter program (https://bibiserv.cebitec.uni-bielefeld.de/reputer/) (Kurtz, 2001). The criteria for repeat determination include a minimum repeat size of 20 bp with a similarity between repeat pairs of 90% by putting edit value 3. Furthermore, MISA software (http://pgrc.ipk-gatersleben.de/misa/) was used to evaluate the simple sequence repeats (SSRs) with the parameters of ‘10’ for mono, ‘5’ for di-, ‘4’ for tri-, and ‘3’ for tetra-, penta-, and hexanucleotide motifs (Beier et al., 2017).

### Comparative and phylogenetic analyses

Relative synonymous codon usage (RSCU) and codon usage values were analyzed by CodonW v.1.4.2. Moreover, the RSCU values were shown in a heatmap by Tbtools (Chen et al., 2020). The contraction and expansion of IR regions at the junctions were visualized using the online IRscope (https://irscope.shinyapps.io/irapp/) (Amiryousefi et al., 2018). The mVISTA program in Shuffle LAGAN mode was used to compare the cp genomes of seven species of *Isodon* (Frazer et al., 2004), using the annotation information of *I. serra* (GenBank NC064127) as a reference. Additionally, the nucleotide variability (Pi) across the cp genome sequences was assessed using DnaSP v.6.12.03, with a window length of 600 sites and a step size of 200 sites (Rozas et al., 2017). A value of Pi higher than 0.014 was recommended as mutational hotspots (Cui et al., 2019a). In phylogenetic analyses, 36 species, including 29 species downloaded from NCBI (**Table S2**), were used to infer the phylogeny. Simultaneously, two species, Scrophularia dentata (GenBank NC036942) and S. henryi (GenBank NC036943) were used as outgroups (Chase et al., 2016). All these sequences were aligned using MAFFT, and alignments were checked manually. The Maximum-likelihood (ML) tree was reconstructed with an IQtree using default parameters of 1000 iterations, 1000 replications, and best-fit model selection (Nguyen et al., 2015).

## Results and discussion

### Genome structure

**Figure 1:**
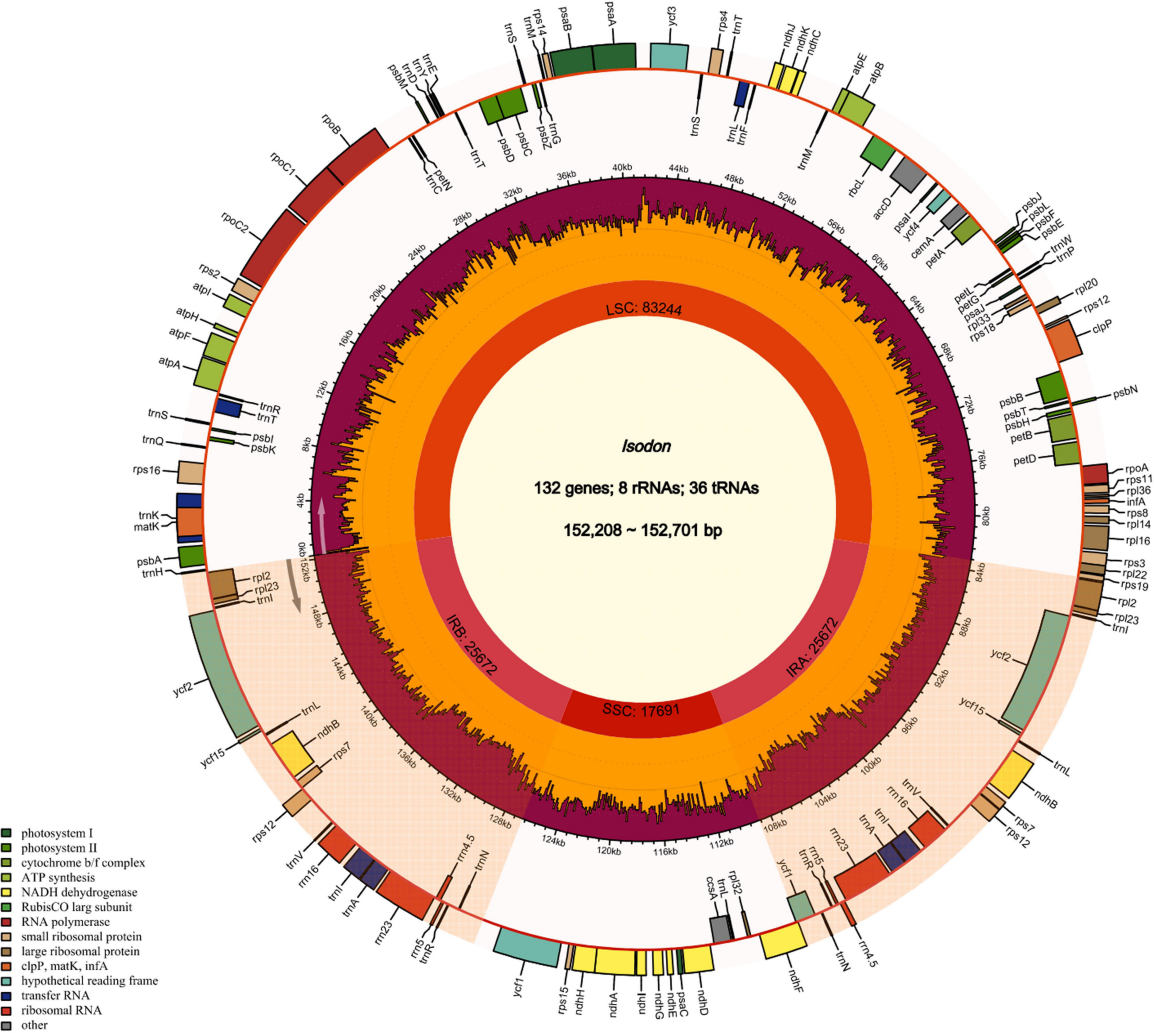
Cp genome map of *Isodon.*

The cp genome size and gene content of seven *Isodon* species are listed in **Table 1**. All the genome sizes were similar to that of the reference genome, i.e., around 150 kb. *I. inflexu* had the largest genome, with a size of 152,701 bp, and *I. japonicus* had the smallest genome, with a size of 152,208 bp. Moreover, the length of large single copy (LSC) regions ranged from 83,079 bp (*I. lophanthoides*) to 83,577 bp (*I. rubescens*), small single copy (SSC) regions ranged from 17,656 bp (*I. excisus*) to 17,729 bp (*I. lophanthoides*), and IRa and IRb regions ranged from 25,700 bp (*I. lophanthoides* and *I. japonicus*) to 25,728 bp (*I. excisus*). In addition, the cp genomes of *Isodon* contained 132 genes, including 88 protein-coding genes, 8 rRNA genes, and 36 tRNA genes, 18 of which are repeated as members of IR regions (**Figure 1**), which were congruent and largely concordant with recent studies of *Isodon* (Yue et al., 2021; Lian et al., 2022). It is worth noting that the *chIB*, *chIL*, and *ycf68* were lost during evolution, typically in most angiosperms (Millen et al., 2001). Meanwhile, the number and types of introns were similar among the seven *Isodon* species (**Table S3**). Each of the 18 genes contained one intron, including *trnA-UGC* (×2), *trnI-GAU* (×2), *rpl2* (×2), and *ndhB* (×2) were located in the IR, and the genes (*trnK-UUU*, *rps16*, *trnT-CGU*, *atpF*, *rpoC1*, *trnL-UAA*, *petB*, *petD*, and *rpl16*) were located in the LSC, and the *ndhA* was the only present in the SSC region. Furthermore, *ycf3* and *clpP* have two introns, respectively, consistent with previous genetic studies (Jiang et al., 2022). The GC content ranged from 37.60% to 37.64% and varied among the different regions. The findings were identical to those of other *Isodon* species (Yue et al., 2021), which is not unexpected, given that the angiosperms possess the highly conserved cp character at the genus level (Meng et al., 2018; Cui et al., 2019b; Shahzadi et al., 2020).

### Codon usage bias of cp genomes

**Figure 2:**
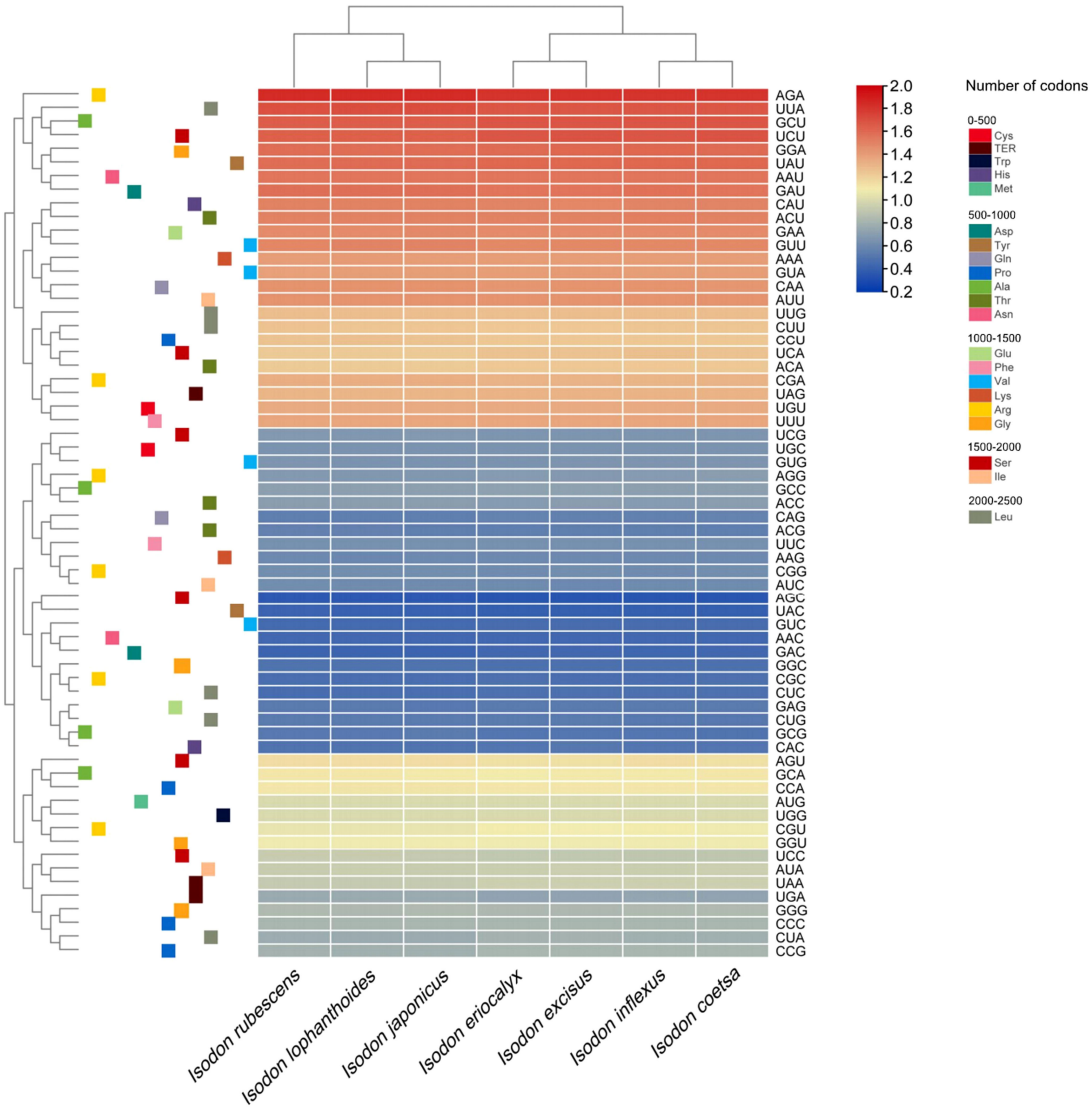
Heat map of the RSCU values among *Isodon* cp genome.

The analyses of relative synonymous codon usage (RSCU) provide information about the encoding frequency of codons for an amino acid. As shown in **Table S4**, the results of RSCU revealed that the cp protein sequences encoded 21 amino acids, and 30 codons were used frequently in *Isodon* species, consistent with recent codon usage studies (Lian et al., 2022). Moreover, amino acid frequency analyses confirmed the highest frequency of leucine and isoleucine, whereas cysteine was a rare amino acid (**Table S4**), which was supported by other researchers based on codon usage bias (Lian et al., 2022). Notably, the use of start codon AUG for methionine and UGG for tryptophan in the *Isodon* genus showed no codon usage bias, consistent with previous reports of *Isodon* (Lian et al., 2022). In general, we found high similarities in codon usage and amino acid frequency among the seven species of *Isodon*. Furthermore, as illustrated in **Figure 2**, higher RSCU values (≥1) were found for codons with A or T at the 3’ position, which showed high encoding efficacy. Similar findings were reported for codon usage and amino acid frequency in the cp genomes of other angiosperms, which may be attributable to the high overall AT content in the cp genome (Kawabe and Miyashita, 2003; Jiang et al., 2022; Wang et al., 2022). In addition, the codon usage was similar in *I. rubescens*, *I. lophanthoides*, and *I. japonicaus*, whereas the codon usage of *I. eriocalyx* and *I. excisus* was relatively close to that of *I. inflexus* and *I. coetsa* (**Figure 2**).

It was shown that the GC content of synonymous third codons positions (GC3s) was closely related to codon bias, which provided the foundation for assessing the codon usage pattern (Shang et al., 2011). In our study, the values of GC3s ranged from 29.4% to 29.5%, demonstrating that the genus *Isodon* had a greater preference for the A/U ending codons, which, along with the highly conserved GC content in seven *Isodon* cp genomes, suggest that natural selection had a profound impact on codon usage patterns (Zhang et al., 2018). In addition, the values for the effective number of codons ranged from 51.73 to 51.81, and both the codon adaptation index and frequency of optimal were less than 0.5. These findings indicated a slight bias of codon usage in the seven *Isodon* species.

### Repeat analysis

**Figure 3:**
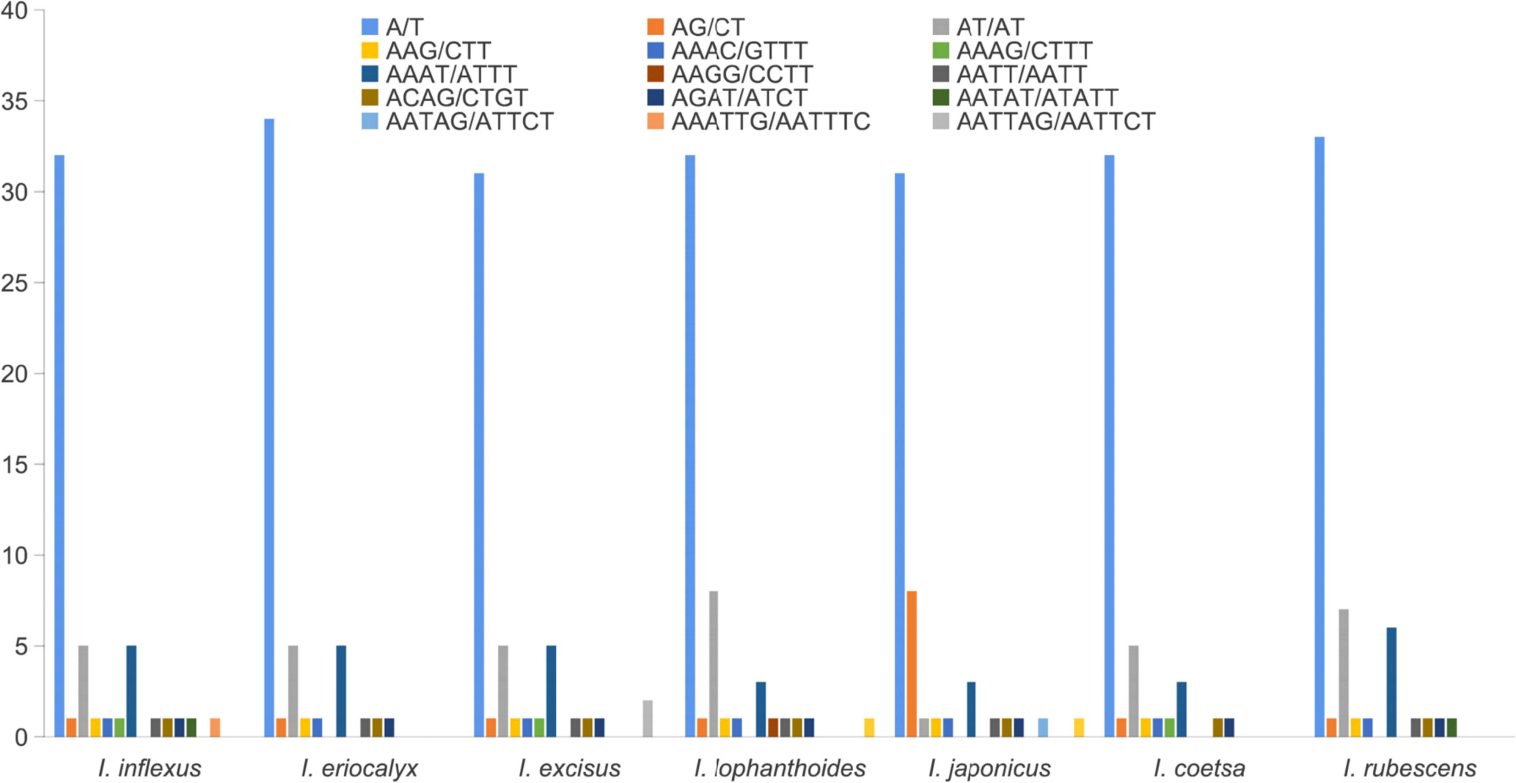
The number and type of SSRs in the cp genome of *Isodon.*

Repetitive sequences play a crucial role in the rearrangement and stability of cp genomes (Rahemi et al., 2012; Kumar et al., 2015). A total of 51, 50, 50, 51, 50, 46, and 53 SSRs were identified in *I. inflexus*, *I. eriocalyx*, *I. excisus*, *I. lophanthoides*, *I. japonicus*, *I. coetsa*, and *I. rubescens*, respectively (**Figure 3**). More than half of SSRs (62.00% – 69.60%) were mononucleotide A/T motifs, which is consistent with the previous works in the cp genomes of angiosperms (Qian et al., 2013; Liang et al., 2019). The second was dinucleotide (11.65% – 18.00%) with a predominant motif of AT/TA, followed by tetranucleotide repeats (14.00% – 19.61%) with a predominant motif of AAAT/ATTT and AATC/ATTG, trinucleotide (1.89% – 2.17%), pentanucleotide (1.89% – 2.00%) with a predominant motif of AATAT/ATATT, AATAG/ATTCT. Hexanucleotides (1.96% – 4.00%) were absent in the cp genomes of *I. eriocalyx*, *I. coetsa*, and *I. rubescens.* This result was consistent with previous findings that most SSRs include mono- and dinucleotide repeats, while tri-, tetra-, penta-, and hexanucleotide repeat sequences exhibit lower frequencies (Dong et al., 2018).

**Figure 4:**
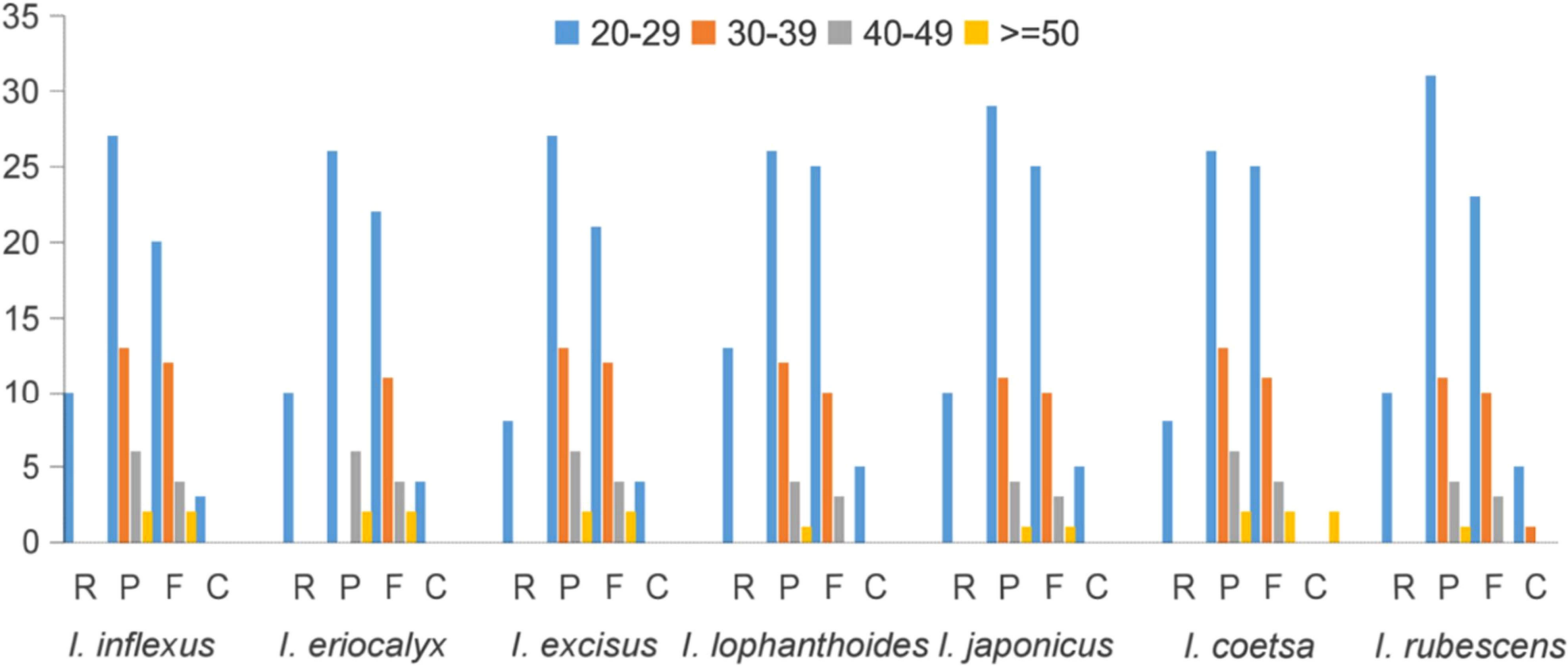
Repeat sequences detected in *Isodon* cp genome. P, F, C, and R indicate the repeat types: R (Reverse repeats), P (Palindromic repeats), F (Forward repeats), C (Complement repeats).

Moreover, oligonucleotide repeats analysis of four types of repeats in the cp genome, including F, R, P, and C, was performed by REPuter. As illustrated in **Figure 4**, the number of repeat types varied and presented random permutations among the cp genomes of seven species. The number of repeats varied among these species, but most repeat sequences existed in 20 – 29 bp, which was supported by recent literature (Lian et al., 2022). Meanwhile, the abundance of F and P repeats was higher than that of R and C repeats. A total of 36 F repeats and 47 P repeats were observed in *I. rubescens*, 38 and 48 in *I. inflexus*, 34 and 39 in *I. eriocalyx*, 39 and 48 in *I. excisus*, 38 and 43 in *I. lophanthoides*, 39 and 45 in *I. japonicus*, 42 and 47 in *I. coetsa*, respectively. These repeats play a crucial role in the generation of substitutions and indels, which makes them important for detecting mutational hotspots (McDonald et al., 2011; Ahmed et al., 2012)

### Inverted repeats

**Figure 5:**
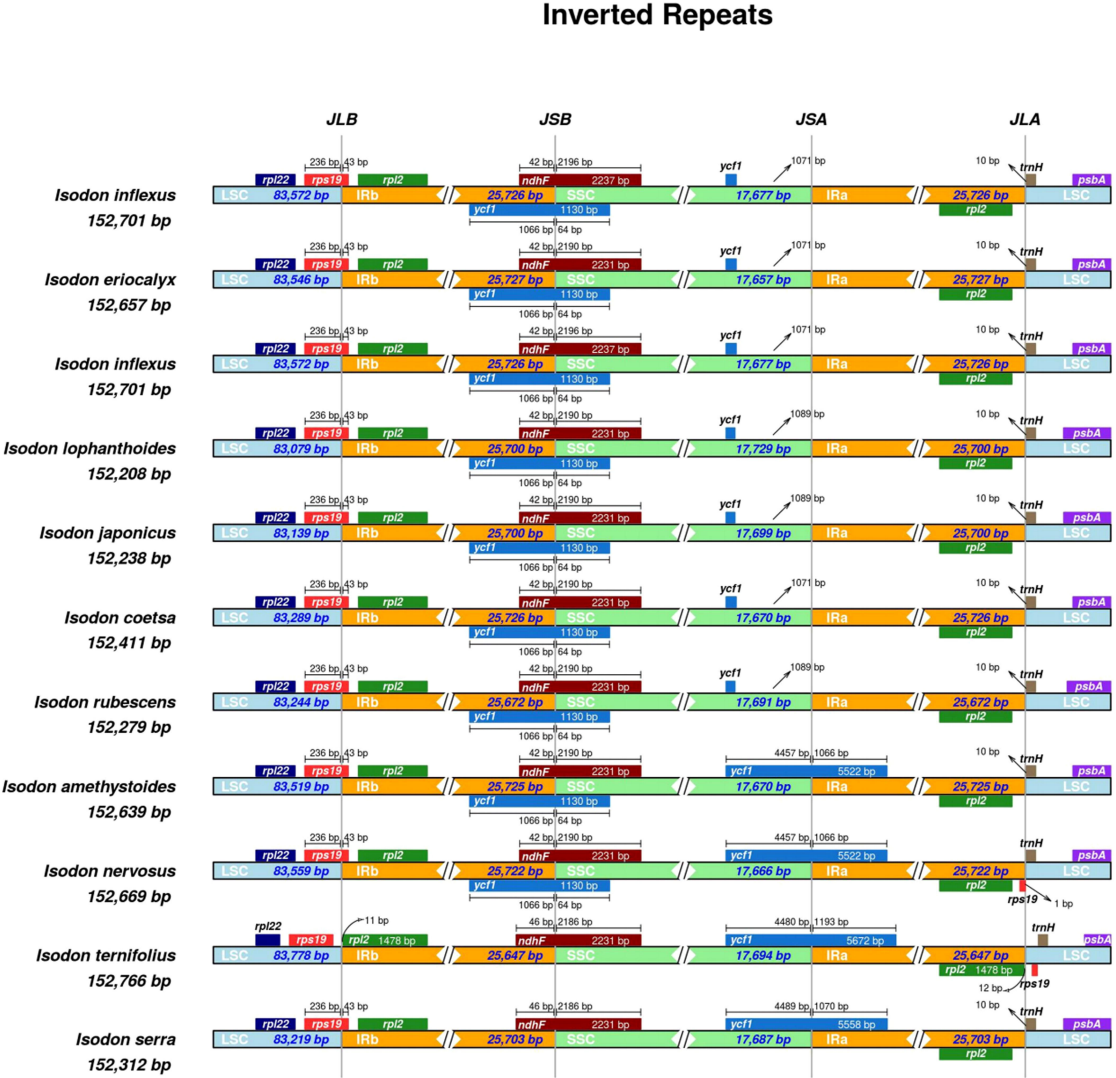
Comparisons of the borders of LSC, SSC, and IRa/b regions among the 11 *Isodon* plastid genomes. The numbers represent the distance between the gene ends and the border sites, and the numbers below represent the length of the LSC, SSC, and IRa/b regions. This Figure is not to scale.

The contraction and expansion of IRs are regarded as crucial evolutionary phenomena that result in the pseudogenes, gene duplication, or the reduction of duplicate genes to a single copy (Abdullah et al., 2020). As illustrated in **Figure 5**, the *rpl22* gene was present in the LSC region, and *rpl2* was entirely in the IRb region, consistent with a previous study (Lian et al., 2022). A truncated copy of *rps19* gene was observed in all species at the IRb/LSC junction except for *I. ternifolious*, which starts in LSC and integrates into the IRb regions, while *rp12* is exclusively located in the IRb region. In monocotyledons, the *rps19* gene is in the IR region (Ahmed et al., 2012; Henriquez et al., 2020), but our findings show that things are different in Lamiaceae. Additionally, the *ndhF* gene was also found at the junction of IRb/SSC and integrated into the IRb with a size ranging from 42 to 46 kb. At the IRb/LSC junction, another truncated copy of *ycf1* gene was observed in all species except for *I. ternifolious* and *I. serra*. Contrary to the findings of Henriquez et al. (2020), the *de novo* assembled genomes of seven *Isodon* species did not include a *ycf* pseudogene at the IRa/SSC junction. Neither an internal stop codon nor double peaks were observed in the electropherograms, indicating that the seven *Isodon* species lack amplified pseudogenes. In addition, *psbA* and *trnH* were in the LSC, and *rpl2* in the IRa.

### Genome comparison and nucleotide diversity

**Figure 6:**
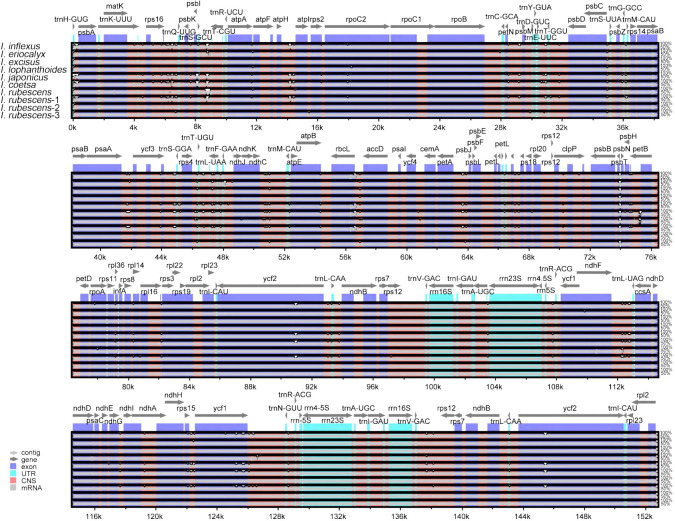
Global comparison of complete genomes of *Isodon.*

**Figure 7:**
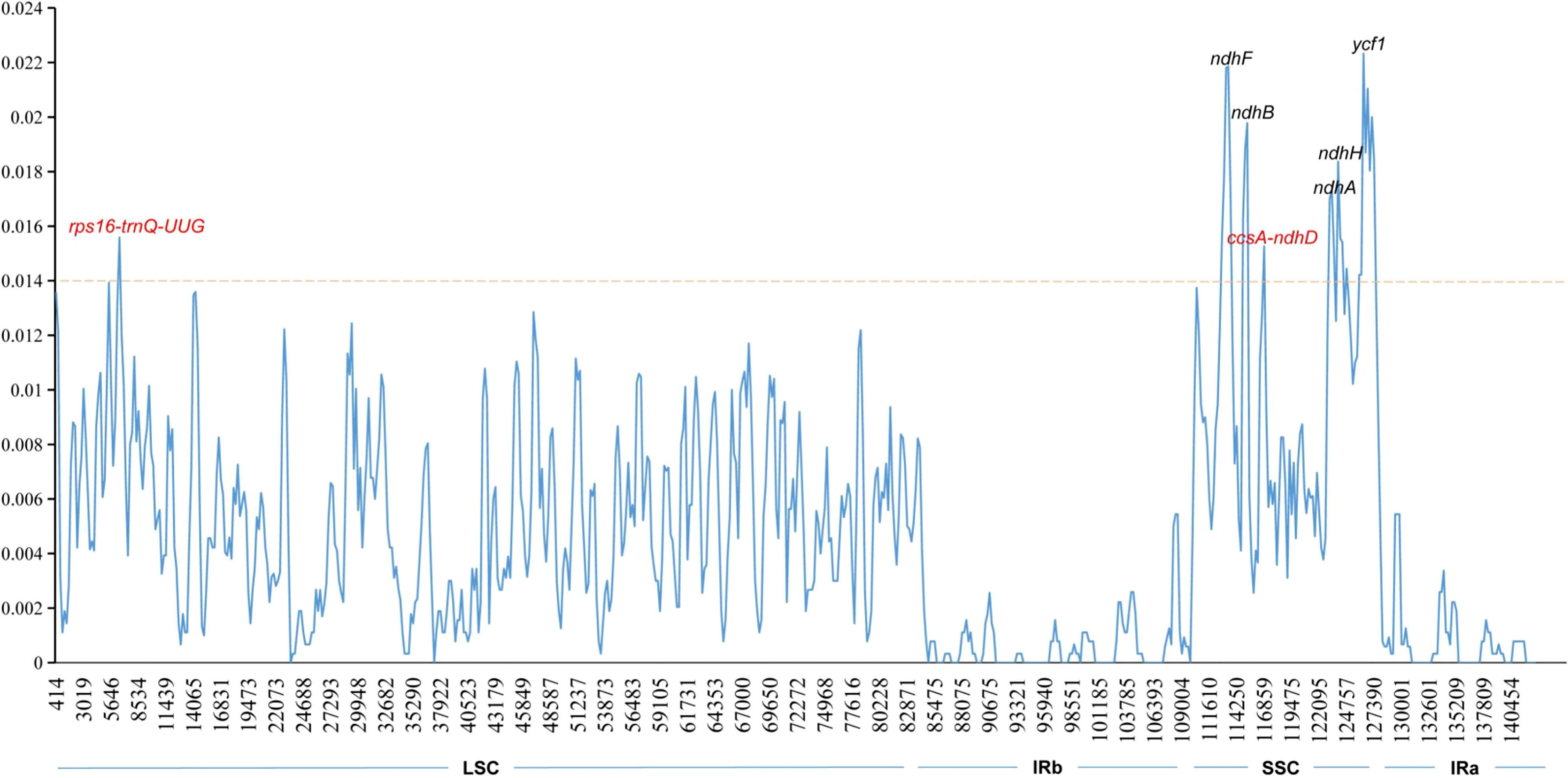
Sliding window analysis of *Isodon* cp genome. The X-axis represents the midpoint of the window; The Y-axis represents nucleotide diversity values. Window length: 600 bp; step size: 200 bp.

A comparison of overall sequence variation showed that the cp genome of *Isodon* is highly conserved, and the coding region is more conserved than non-coding regions. Except for *ndhF*, *ycf1*, and *ycf2* genes, all protein-coding genes showed a highly conserved character (**Figure 6**); the intergenic spacers (IGS) with the highest divergence were *trnH-GUG-psbA*, *trnQ-UUG-psbK*, *trnS-GCU-trnT-CGU*, *atpH-atpI*, *trnE-UUC-trnT-GGU, psaA-ycf3, ndhC-trnM-CAU, psbH-petB, and rps15-ycf1*, as predicted. In addition, the sliding window analysis revealed that seven regions, including *rps16-trnQ-UUG*, *ndhF*, *ndhB*, *ccsA-ndhD*, *ndhA*, *ndhH*, and *ycf1* genes, exhibited higher nucleotide diversity values (> 0.014, **Figure 7**); the IR regions exhibited lower sequence divergence than LSC and SSC regions, consistent with the previous comparisons of cp genomes (Jiang et al., 2022; Wang et al., 2022). Among these 16 high polymorphic regions, 11 intergenic spacers were found in the *trnH-GUG-psbA*, *trnQ-UUG-psbK*, *trnS-GCU-trnT-CGU*, *atpH-atpI*, *trnE-UUC-trnT-GGU, psaA-ycf3, ndhC-trnM-CAU, psbH-petB, rps15-ycf1*, *rps16-trnQ-UUG*, and *ccsA-ndhD*. It is noteworthy that the *atpH-atpI*, *rps16-trnQ-UUG*, and *ndhC-trnM-CAU* were also identified as mutational hotspots in a previous study (Lian et al., 2022). IGS was considered one of the evolutionary hotspots that exhibited more significant rates of nucleotide substitutions and indel mutations (Drummond, 2008). Therefore, these IGS might be undergoing more rapid nucleotide substitution at the species level, which could be served as a potential molecular marker for application in phylogenetic analyses of the *Isodon* genus.

### Species authentication analysis based on IGS

Intergenic spacer regions are the most frequently used cp markers for phylogenetic studies at lower taxonomic levels in plants (Shaw et al., 2005), as they are regarded as more variable and could provide more phylogenetically informative characters. To find candidate markers for identifying *I. rubescens* and its adulterants, the 11 IGSs were extracted using the PhyloSuite v1.2.2 from 14 *Isodon* species (Zhang et al., 2019). Each of the 11 IGSs was subject to maximum likelihood analyses in IQtree (Nguyen et al., 2015). As illustrated in **Figure S2.1**-**S2.11**, five IGSs, including *trnS-GCU-trnT-CGU*, *atpH-atpI*, *trnE-UUC-trnT-GGU*, *ndhC-trnM-CAU*, and *rps15-ycf1* could be distinguished *I. rubescens* from *I. japonicus* and *I. lophanthoides*. Whereas the rest of the *Isodon* species cannot be distinguished based on these IGSs, bootstrap values for the relationship among these major clades were weak (<70%). These results are partially in line with the previous study, which found that the *atpH-atpI* and *ndhC-trnM-CAU* could be potential molecular markers for distinguishing *Isodon* species (Lian et al., 2022). Moreover, the remaining fragment, including *trnS-GCU-trnT-CGU, trnE-UUC-trnT-GGU*, and *rps15-ycf1* also reported as potential markers for other species identification (Kim et al., 2020; Li et al., 2020; Alzahrani, 2021). Although the previous study has revealed that universal DNA barcodes (e.g., *psbA-trnH*) could differentiate *I. rubescens* from their related species (Xia et al., 2013), some common adulterants were not included in this study. Furthermore, the comparative analysis showed that the screened IGSs exhibit higher variability than *psbA-trnH*. These IGSs could theoretically distinguish the selected 7 species, whereas a much more detailed investigation of identification accuracy and amplification efficiency needs to be accomplished, and more experimental evidence is needed. Moreover, the ML phylogenetic tree was also inferred using a combination of these five IGSs. The results (**Figure S3**) showed that *I. rubescens* (GenBank NW018469) and *I. rubescens* (GenBank NC053708) clustered together as sister group groups and closely related to *I. excisus*. Simultaneously, *I. rubescens* (GenBank OM808736) was located in independent branches in the phylogeny, and well-supported sister relationship between *I. rubescens* and *I. japonicus* + *I. lophanthoides* (100% B/S). These results indicated that the combination of five IGSs could effectively discriminate *I. rubescens* from its common adulterants.

### Species identification and phylogenetic analysis

**Figure 8:**
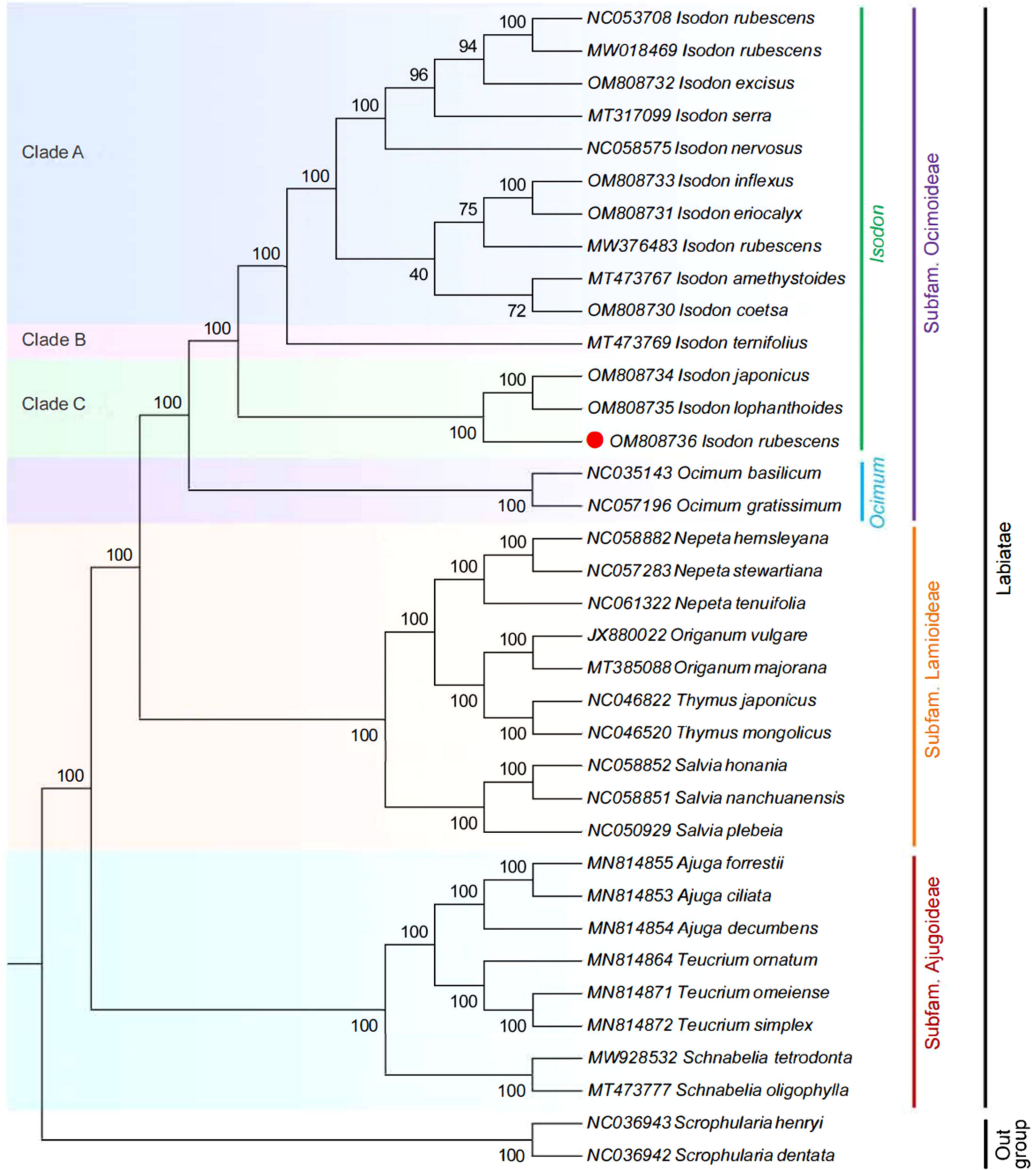
ML phylogenetic tree reconstruction containing the cp genomes of 36 plants. The *Sceophularia* species were set as the outgroup.

The ML phylogenetic tree was inferred using 36 species, with *Scrophularia* as the outgroup. As illustrated in **Figure 8**, on the consensus trees, most nodes were supported with maximum support (100% bootstrap support). Ocimoideae and Lamioideae were sister taxa within the three subfamilies, and Ajugoideae was sister to the clade containing Ocimoideae + Lamioideae. The phylogenetic tree’s crown was occupied by the subfamily Ocimoideae, which included the genera *Isodon* and *Ocimum*. These findings confirm the position of *Isodon* within the Lamiaceae and are consistent with previous phylogenomic studies (Chen et al., 2021; Yue et al., 2021). The genus *Isodon* is further divided into three clades: (i) clade A, including *I. rubescens* (GenBank NC053708, GenBank NW018469), *I. excise*, *I. serra*, *I. nervosus*, *I. amethystoides*, *I. coetsa*, *I. inflexus*, *I. eriocalyx*, and *I. rubescens* (GenBank NW376483); (ii) clade B, only *I. ternifolious*; (iii) clade C, including *I. japonicas*, *I. lophanthoides*, and *I. rubescens* (GenBank OM808736). It is worth noting that the *I. rubescens* (GenBank OM808736) clustered differently from the other three samples (GenBank NC053708, GenBank NW018469, GenBank NW376483) of the same species in the phylogeny, which is consistent with the finding of other researcher based on *rps16*, *trnL-trnF*, and ITS sequence (Harris et al., 2012). Moreover, Lian et al. (2022) also found that the samples of *I. rubescens* from different geographical areas were not recovered as monophyletic and were placed in different branches in the previous report, which is well distinguished by sampling locations, suggesting that the intraspecific diversity was present in *I. rubescens.* This phenomenon might be explained by the fact that the geographical area of origin might influence the variation of *I. rubescens*. Another previous study supported the same conclusion; the *Artemisia argyi* collected from different geographical areas display high intraspecific diversity in the cp genome (Chen et al., 2022). In addition, clade B only includes a species of *I. ternifolious*, which was supported by the findings of other researchers based on *trnD-trnT*, *psbA-trnH*, *rpl32-trnL*, *trnL-trnF*, *rps16*, and *nr*ITS (Zhong et al., 2010; Yu et al., 2014; Chen et al., 2021). Moreover, maximum likelihood analysis demonstrated that *I. rubescens* (GenBank OM808736) was located in independent branches in the phylogeny and deeply nested within clade C, and the sister relationship between *I. rubescens* and *I. japonicus* + *I. lophanthoides* was highly supported (100% B/S), indicating that the cp genome could discriminate *I. rubescens* from its common adulterants. The findings supported the results of morphological classification reported by Ge et al. (2022) and Xia et al. (2013).

## Conclusion

In the current study, the complete cp genome of seven species of *Isodon* was *de novo* assembled from Illumina high throughput sequencing reads, and cp genome sequences of *I. inflexus*, *I. eriocalyx*, *I. excisus*, and *I. coetsa* were reported for the first time. These cp genomes were generally conserved and exhibited similar gene content and genomic structure. Five highly variable cp loci, including *trnS-GCU-trnT-CGU*, *atpH-atpI*, *trnE-UUC-trnT-GGU, ndhC-trnM-CAU*, and *rps15-ycf1*, were identified, which could serve as potential markers for identifying *I. rubescens* and its common adulterants. In conclusion, our study provides a powerful tool and valuable scientific reference for the safety and effectiveness of clinical drug use, and it also contributes to the bioprospecting and conservation of *Isodon* species.

## Data availability statement

The data presented in the study are deposited in the GenBank repository, accession numbers were from OM808730 to OM808736.

## Author contributions

ZZ, JW, TP, and BD participated in the conception and design of the research. JW, QG, and BD collected the species. JQ, LS, and JD are responsible for analyzing and processing data. ZZ wrote the manuscript. The paper was revised by JQ, LS, and BD. All authors contributed to the article and approved the submitted version.

## Funding

This work was supported by the Yunnan academician expert workstation (202205AF150026, 202105AF150053), the key technology projects in the Yunnan province of China (202002AA100007), and the Yunnan Xingdian talent support plan (YNWR-QNBJ-2020251).

## Acknowledgments

We would like to thank Ms. Qingshu Yang for her assistance in obtaining specimens for this study. We also thank Northeast Forestry University and the China Academy of Chinese Medical Sciences for technical assistance.

## Conflict of interest

The authors declare that the research was conducted in the absence of any commercial or financial relationships that could be construed as a potential conflict of interest.

## Publisher’s note

All claims expressed in this article are solely those of the authors and do not necessarily represent those of their affiliated organizations, or those of the publisher, the editors and the reviewers. Any product that may be evaluated in this article, or claim that may be made by its manufacturer, is not guaranteed or endorsed by the publisher.
