# Supplementary material for: Comparative analysis of medicinal plant Isodon rubescens and its common adulterants based on chloroplast genome sequencing
Source: Front Plant Sci. 2022 Nov 21;13:1036277. doi: 10.3389/fpls.2022.1036277 (PMC9720329; doi:10.3389/fpls.2022.1036277)
Supplement: Supplementary file 1 [file DataSheet_1.docx]

Table S1 Information about the samples collected

| No. | Species | Locality | Voucher specimen |
| --- | --- | --- | --- |
| 1 | *I. inflexus* | Kunming, Yunnan, China | XC20210701 |
| 2 | *I. eriocalyx* | Kunming, Yunnan, China | XC20210702 |
| 3 | *I. excisus* | Kunming, Yunnan, China | XC20210703 |
| 4 | *I. lophanthoides* | Kunming, Yunnan, China | XC20210704 |
| 5 | *I. japonicus* | Kunming, Yunnan, China | XC20210705 |
| 6 | *I. coetsa* | Kunming, Yunnan, China | XC20210706 |
| 7 | *I. rubescens* | Kunming, Yunnan, China | XC20210707 |

Table S2 Species information downloaded by NCBI

| No. | Species | GenBank accession | No. | Species | GenBank accession |
| --- | --- | --- | --- | --- | --- |
| 1 | *[Isodon rubescens](https://www.ncbi.nlm.nih.gov/nuccore/NC_053708.1)* | NC053708 | 16 | *Thymus mongolicus* | NC046520 |
| 2 | *[Isodon rubescens](https://www.ncbi.nlm.nih.gov/nuccore/NC_053708.1)* | MW018469 | 17 | *Salvia honania* | NC058852 |
| 3 | *[Isodon rubescens](https://www.ncbi.nlm.nih.gov/nuccore/NC_053708.1)* | MW376483 | 18 | *Salvia nanchuanensis* | NC058851 |
| 4 | *Isodon nervosus* | NC058575 | 19 | *Salvia plebeia* | NC050929 |
| 5 | *Isodon serra* | MT317099 | 20 | *Ajuga forrestii* | MN814855 |
| 6 | *Isodon amethystoides* | MT473767 | 21 | *Ajuga ciliata* | MN814853 |
| 7 | *Isodon ternifolius* | MT473769 | 22 | *Ajuga decumbens* | MN814854 |
| 8 | *Ocimum basilicum* | NC035143 | 23 | *Teucrium ornatum* | MN814864 |
| 9 | *Ocimum gratissimum* | NC057196 | 24 | *Teucrium omeiense* | MN814871 |
| 10 | *Nepeta hemsleyana* | NC058882 | 25 | *Teucrium simplex* | MN814872 |
| 11 | *Nepeta stewartiana* | NC057283 | 26 | *Schnabelia tetrodonta* | MW928532 |
| 12 | *Nepeta tenuifolia* | NC061322 | 27 | *Schnabelia oligophylla* | MT473777 |
| 13 | *Origanum vulgare* | JX880022 | 28 | *Scrophularia henryi* | NC036943 |
| 14 | *Origanum majorana* | MT385088 | 29 | *Scrophularia dentata* | NC036942 |
| 15 | *Thymus japonicus* | NC046822 |  |  |  |

Table S3 Gene composition in *Isodon* cp genome

| Category for genes | Group of genes | Name of genes |
| --- | --- | --- |
| Self replication | Ribosomal RNAs | *rrn16S*(×2)、rrn*23S*(×2)、rrn*4.5S*(×2)、rrn*5S*(×2) |
|  | Transfer RNAs | *trnA-UG*C(×2)*、trnC-GCA、trnD-GUC、trnE-UUC、trnF-GAA、trnG-UCC*、*trnH-GUG*、*trnI-CAU*(×2)、*trnI-GAU*(×2)、*trnK-UUU**、*trnL-CAA*(×2)*、trnL-UAA***、trnL-UAG、trnM-CAU*、*trnN-GUU*、*trnP-UGG、trnQ-UUG、trnR-ACG*(×2)*、trnR-UCU、trnS-GCU、trnS-GGA、trnS-UGA、trnT-GGU、trnT-UGU、trnV-GAC*(×2)*、trnV-UAC*、*trnW-CCA、trnY-GUA* |
|  | Small subunit of ribosome | *rps11、rps12*(×2)、rps*14*、rps*15*、rps*16**、rps*18*、rps*19*、rps*2*、rps*3*、rps*4*、rps*7*(×2)、rps*8* |
|  | Large subunit of ribosome | *rpl14、rpl16、rpl2*(×2)**、rpl20、rpl22、rpl23*(×2)*、rpl32、rpl33、rpl36* |
|  | RNA polymerase | *rpoA、rpoB、rpoC1、rpoC2* |
| Genes for  photosynthesis | PhotosystemI | *psaA、psaB、psaC、psaI、psaJ* |
|  | PhotosystemⅡ | *psbA、psbB、psbC、psbD、psbE、psbF、psbI、psbJ、psbK、psbL、psbM、psbN、psbT、psbZ、ycf3* |
|  | Cytochrome b/f complex | *petA、petB***、petD***、petG、petL、petN* |
|  | ATP synthase | *atpA、atpB、atpE、atpF***、atpH、atpI* |
|  | Rubisco | *rbcL* |
|  | NADH dehydrogenase | *ndhA***、ndhB*(×2)*、ndhC、ndhD、ndhE、ndhF、ndhG、ndhH、ndhI、ndhJ、ndhK* |
| Other genes | Maturase | *matK* |
|  | Envelop membrane protein | *cemA* |
|  | acetyl-CoA-carboxylase | *accD* |
|  | c-type cytochrome synthesis | *ccsA* |
|  | Protease | *clpP** |
| Genes of unknown function | Conserved open reading frames | *ycf1*(×2)、*ycf15*(×2)、*ycf2*(×2)*、*ycf4* |

Table S4.1 Codons in the cp genome of *I. inflexus*

| Amino acid | Codon | No. | RSCU | Amino acid | Codon | No. | RSCU | Amino acid | Codon | No. | RSCU | Amino acid | Codon | No. | RSCU |
| --- | --- | --- | --- | --- | --- | --- | --- | --- | --- | --- | --- | --- | --- | --- | --- |
| Phe | UUU | 757 | 1.37 | Ser | UCU | 441 | 1.68 | Tyr | UAU | 613 | 1.61 | Cys | UGU | 176 | 1.35 |
|  | UUC | 347 | 0.63 |  | UCC | 236 | 0.9 |  | UAC | 149 | 0.39 |  | UGC | 85 | 0.65 |
| Leu | UUA | 678 | 1.68 |  | UCA | 333 | 1.27 | TER | UAA | 98 | 0.95 | TER | UGA | 76 | 0.74 |
|  | UUG | 516 | 1.28 |  | UCG | 173 | 0.66 |  | UAG | 136 | 1.32 | Trp | UGG | 341 | 1 |
|  | CUU | 503 | 1.25 | Pro | CCU | 262 | 1.24 | His | CAU | 328 | 1.5 | Arg | CGU | 214 | 1.1 |
|  | CUC | 194 | 0.48 |  | CCC | 173 | 0.82 |  | CAC | 110 | 0.5 |  | CGC | 92 | 0.47 |
|  | CUA | 321 | 0.79 |  | CCA | 237 | 1.12 | Gln | CAA | 569 | 1.44 |  | CGA | 256 | 1.31 |
|  | CUG | 211 | 0.52 |  | CCG | 174 | 0.82 |  | CAG | 224 | 0.56 |  | CGG | 119 | 0.61 |
| Ile | AUU | 857 | 1.45 | Thr | ACU | 351 | 1.52 | Asn | AAU | 739 | 1.56 | Ser | AGU | 303 | 1.16 |
|  | AUC | 362 | 0.61 |  | ACC | 159 | 0.69 |  | AAC | 209 | 0.44 |  | AGC | 88 | 0.34 |
|  | AUA | 558 | 0.94 |  | ACA | 286 | 1.24 | Lys | AAA | 822 | 1.4 | Arg | AGA | 353 | 1.81 |
| Met | AUG | 503 | 1 |  | ACG | 126 | 0.55 |  | AAG | 351 | 0.6 |  | AGG | 135 | 0.69 |
| Val | GUU | 436 | 1.48 | Ala | GCU | 374 | 1.68 | Asp | GAU | 595 | 1.57 | Gly | GGU | 330 | 1.08 |
|  | GUC | 136 | 0.46 |  | GCC | 158 | 0.71 |  | GAC | 165 | 0.43 |  | GGC | 148 | 0.48 |
|  | GUA | 413 | 1.4 |  | GCA | 246 | 1.11 | Glu | GAA | 773 | 1.47 |  | GGA | 492 | 1.61 |
|  | GUG | 193 | 0.66 |  | GCG | 111 | 0.5 |  | GAG | 280 | 0.53 |  | GGG | 256 | 0.84 |

Table S4.2 Codons in the cp genome of *I. eriocalyx*

| Amino acid | Codon | No. | RSCU | Amino acid | Codon | No. | RSCU | Amino acid | Codon | No. | RSCU | Amino acid | Codon | No. | RSCU |
| --- | --- | --- | --- | --- | --- | --- | --- | --- | --- | --- | --- | --- | --- | --- | --- |
| Phe | UUU | 755 | 1.37 | Ser | UCU | 441 | 1.68 | Tyr | UAU | 614 | 1.61 | Cys | UGU | 176 | 1.35 |
|  | UUC | 346 | 0.63 |  | UCC | 238 | 0.91 |  | UAC | 149 | 0.39 |  | UGC | 85 | 0.65 |
| Leu | UUA | 680 | 1.68 |  | UCA | 331 | 1.26 | TER | UAA | 99 | 0.95 | TER | UGA | 76 | 0.73 |
|  | UUG | 512 | 1.27 |  | UCG | 173 | 0.66 |  | UAG | 137 | 1.32 | Trp | UGG | 341 | 1 |
|  | CUU | 503 | 1.24 | Pro | CCU | 262 | 1.24 | His | CAU | 329 | 1.5 | Arg | CGU | 213 | 1.1 |
|  | CUC | 193 | 0.48 |  | CCC | 173 | 0.82 |  | CAC | 110 | 0.5 |  | CGC | 93 | 0.48 |
|  | CUA | 323 | 0.8 |  | CCA | 237 | 1.12 | Gln | CAA | 568 | 1.43 |  | CGA | 256 | 1.32 |
|  | CUG | 214 | 0.53 |  | CCG | 174 | 0.82 |  | CAG | 225 | 0.57 |  | CGG | 119 | 0.61 |
| Ile | AUU | 859 | 1.45 | Thr | ACU | 348 | 1.51 | Asn | AAU | 739 | 1.56 | Ser | AGU | 300 | 1.15 |
|  | AUC | 361 | 0.61 |  | ACC | 159 | 0.69 |  | AAC | 208 | 0.44 |  | AGC | 88 | 0.34 |
|  | AUA | 557 | 0.94 |  | ACA | 286 | 1.24 | Lys | AAA | 821 | 1.4 | Arg | AGA | 352 | 1.81 |
| Met | AUG | 503 | 1 |  | ACG | 127 | 0.55 |  | AAG | 351 | 0.6 |  | AGG | 134 | 0.69 |
| Val | GUU | 436 | 1.48 | Ala | GCU | 375 | 1.68 | Asp | GAU | 594 | 1.56 | Gly | GGU | 332 | 1.08 |
|  | GUC | 135 | 0.46 |  | GCC | 160 | 0.72 |  | GAC | 166 | 0.44 |  | GGC | 148 | 0.48 |
|  | GUA | 413 | 1.4 |  | GCA | 246 | 1.1 | Glu | GAA | 772 | 1.47 |  | GGA | 493 | 1.6 |
|  | GUG | 192 | 0.65 |  | GCG | 112 | 0.5 |  | GAG | 281 | 0.53 |  | GGG | 256 | 0.83 |

Table S4.3 Codons in the cp genome of *I. excisus*

| Amino acid | Codon | No. | RSCU | Amino acid | Codon | No. | RSCU | Amino acid | Codon | No. | RSCU | Amino acid | Codon | No. | RSCU |
| --- | --- | --- | --- | --- | --- | --- | --- | --- | --- | --- | --- | --- | --- | --- | --- |
| Phe | UUU | 755 | 1.37 | Ser | UCU | 441 | 1.69 | Tyr | UAU | 610 | 1.61 | Cys | UGU | 176 | 1.35 |
|  | UUC | 348 | 0.63 |  | UCC | 237 | 0.91 |  | UAC | 149 | 0.39 |  | UGC | 84 | 0.65 |
| Leu | UUA | 677 | 1.68 |  | UCA | 330 | 1.26 | TER | UAA | 98 | 0.95 | TER | UGA | 76 | 0.73 |
|  | UUG | 517 | 1.28 |  | UCG | 173 | 0.66 |  | UAG | 137 | 1.32 | Trp | UGG | 341 | 1 |
|  | CUU | 502 | 1.24 | Pro | CCU | 262 | 1.24 | His | CAU | 328 | 1.49 | Arg | CGU | 212 | 1.09 |
|  | CUC | 192 | 0.48 |  | CCC | 174 | 0.82 |  | CAC | 111 | 0.51 |  | CGC | 92 | 0.47 |
|  | CUA | 323 | 0.8 |  | CCA | 236 | 1.12 | Gln | CAA | 569 | 1.44 |  | CGA | 256 | 1.32 |
|  | CUG | 213 | 0.53 |  | CCG | 174 | 0.82 |  | CAG | 223 | 0.56 |  | CGG | 120 | 0.62 |
| Ile | AUU | 857 | 1.45 | Thr | ACU | 348 | 1.51 | Asn | AAU | 737 | 1.56 | Ser | AGU | 300 | 1.15 |
|  | AUC | 357 | 0.6 |  | ACC | 160 | 0.7 |  | AAC | 209 | 0.44 |  | AGC | 88 | 0.34 |
|  | AUA | 560 | 0.95 |  | ACA | 285 | 1.24 | Lys | AAA | 823 | 1.4 | Arg | AGA | 353 | 1.82 |
| Met | AUG | 503 | 1 |  | ACG | 127 | 0.55 |  | AAG | 352 | 0.6 |  | AGG | 133 | 0.68 |
| Val | GUU | 436 | 1.48 | Ala | GCU | 374 | 1.68 | Asp | GAU | 595 | 1.56 | Gly | GGU | 331 | 1.08 |
|  | GUC | 134 | 0.46 |  | GCC | 160 | 0.72 |  | GAC | 166 | 0.44 |  | GGC | 147 | 0.48 |
|  | GUA | 414 | 1.41 |  | GCA | 247 | 1.11 | Glu | GAA | 772 | 1.47 |  | GGA | 492 | 1.61 |
|  | GUG | 193 | 0.66 |  | GCG | 111 | 0.5 |  | GAG | 281 | 0.53 |  | GGG | 255 | 0.83 |

Table S4.4 Codons in the cp genome of *I. lophanthoides*

| Amino acid | Codon | No. | RSCU | Amino acid | Codon | No. | RSCU | Amino acid | Codon | No. | RSCU | Amino acid | Codon | No. | RSCU |
| --- | --- | --- | --- | --- | --- | --- | --- | --- | --- | --- | --- | --- | --- | --- | --- |
| Phe | UUU | 764 | 1.37 | Ser | UCU | 440 | 1.64 | Tyr | UAU | 620 | 1.6 | Cys | UGU | 181 | 1.35 |
|  | UUC | 352 | 0.63 |  | UCC | 251 | 0.93 |  | UAC | 156 | 0.4 |  | UGC | 87 | 0.65 |
| Leu | UUA | 683 | 1.71 |  | UCA | 331 | 1.23 | TER | UAA | 97 | 0.92 | TER | UGA | 80 | 0.76 |
|  | UUG | 512 | 1.28 |  | UCG | 180 | 0.67 |  | UAG | 138 | 1.31 | Trp | UGG | 333 | 1 |
|  | CUU | 499 | 1.25 | Pro | CCU | 262 | 1.27 | His | CAU | 332 | 1.51 | Arg | CGU | 207 | 1.05 |
|  | CUC | 186 | 0.47 |  | CCC | 169 | 0.82 |  | CAC | 107 | 0.49 |  | CGC | 92 | 0.47 |
|  | CUA | 305 | 0.76 |  | CCA | 233 | 1.13 | Gln | CAA | 568 | 1.45 |  | CGA | 263 | 1.34 |
|  | CUG | 211 | 0.53 |  | CCG | 164 | 0.79 |  | CAG | 215 | 0.55 |  | CGG | 121 | 0.62 |
| Ile | UUU | 764 | 1.37 | Thr | ACU | 352 | 1.51 | Asn | AAU | 741 | 1.56 | Ser | AGU | 312 | 1.16 |
|  | UUC | 352 | 0.63 |  | ACC | 162 | 0.7 |  | AAC | 210 | 0.44 |  | AGC | 97 | 0.36 |
|  | UUA | 683 | 1.71 |  | ACA | 286 | 1.23 | Lys | AAA | 828 | 1.41 | Arg | AGA | 364 | 1.85 |
| Met | UUG | 512 | 1.28 |  | ACG | 130 | 0.56 |  | AAG | 350 | 0.59 |  | AGG | 132 | 0.67 |
| Val | CUU | 499 | 1.25 | Ala | GCU | 374 | 1.65 | Asp | GAU | 594 | 1.58 | Gly | GGU | 333 | 1.08 |
|  | CUC | 186 | 0.47 |  | GCC | 160 | 0.71 |  | GAC | 157 | 0.42 |  | GGC | 151 | 0.49 |
|  | CUA | 305 | 0.76 |  | GCA | 252 | 1.12 | Glu | GAA | 766 | 1.47 |  | GGA | 491 | 1.59 |
|  | CUG | 211 | 0.53 |  | GCG | 118 | 0.52 |  | GAG | 274 | 0.53 |  | GGG | 257 | 0.83 |

Table S4.5 Codons in the cp genome of *I. japonicus*

| Amino acid | Codon | No. | RSCU | Amino acid | Codon | No. | RSCU | Amino acid | Codon | No. | RSCU | Amino acid | Codon | No. | RSCU |
| --- | --- | --- | --- | --- | --- | --- | --- | --- | --- | --- | --- | --- | --- | --- | --- |
| Phe | UUU | 766 | 1.37 | Ser | UCU | 441 | 1.64 | Tyr | UAU | 619 | 1.6 | Cys | UGU | 181 | 1.35 |
|  | UUC | 350 | 0.63 |  | UCC | 248 | 0.92 |  | UAC | 156 | 0.4 |  | UGC | 88 | 0.65 |
| Leu | UUA | 683 | 1.71 |  | UCA | 330 | 1.23 | TER | UAA | 97 | 0.92 | TER | UGA | 80 | 0.76 |
|  | UUG | 512 | 1.28 |  | UCG | 183 | 0.68 |  | UAG | 138 | 1.31 | Trp | UGG | 333 | 1 |
|  | CUU | 500 | 1.25 | Pro | CCU | 261 | 1.26 | His | CAU | 333 | 1.51 | Arg | CGU | 206 | 1.05 |
|  | CUC | 187 | 0.47 |  | CCC | 169 | 0.82 |  | CAC | 107 | 0.49 |  | CGC | 92 | 0.47 |
|  | CUA | 305 | 0.76 |  | CCA | 233 | 1.13 | Gln | CAA | 568 | 1.45 |  | CGA | 264 | 1.34 |
|  | CUG | 211 | 0.53 |  | CCG | 164 | 0.79 |  | CAG | 215 | 0.55 |  | CGG | 121 | 0.62 |
| Ile | AUU | 859 | 1.45 | Thr | ACU | 352 | 1.51 | Asn | AAU | 741 | 1.56 | Ser | AGU | 312 | 1.16 |
|  | AUC | 362 | 0.61 |  | ACC | 162 | 0.7 |  | AAC | 210 | 0.44 |  | AGC | 97 | 0.36 |
|  | AUA | 552 | 0.93 |  | ACA | 287 | 1.23 | Lys | AAA | 826 | 1.4 | Arg | AGA | 363 | 1.85 |
| Met | AUG | 492 | 1 |  | ACG | 129 | 0.55 |  | AAG | 351 | 0.6 |  | AGG | 132 | 0.67 |
| Val | GUU | 430 | 1.5 | Ala | GCU | 374 | 1.65 | Asp | GAU | 595 | 1.58 | Gly | GGU | 332 | 1.08 |
|  | GUC | 128 | 0.45 |  | GCC | 161 | 0.71 |  | GAC | 157 | 0.42 |  | GGC | 152 | 0.49 |
|  | GUA | 402 | 1.4 |  | GCA | 252 | 1.11 | Glu | GAA | 768 | 1.48 |  | GGA | 490 | 1.59 |
|  | GUG | 187 | 0.65 |  | GCG | 119 | 0.53 |  | GAG | 272 | 0.52 |  | GGG | 257 | 0.84 |

Table S4.6 Codons in the cp genome of *I. coetsa*

| Amino acid | Codon | No. | RSCU | Amino acid | Codon | No. | RSCU | Amino acid | Codon | No. | RSCU | Amino acid | Codon | No. | RSCU |
| --- | --- | --- | --- | --- | --- | --- | --- | --- | --- | --- | --- | --- | --- | --- | --- |
| Phe | UUU | 756 | 1.37 | Ser | UCU | 442 | 1.69 | Tyr | UAU | 612 | 1.61 | Cys | UGU | 176 | 1.35 |
|  | UUC | 346 | 0.63 |  | UCC | 237 | 0.9 |  | UAC | 150 | 0.39 |  | UGC | 84 | 0.65 |
| Leu | UUA | 679 | 1.68 |  | UCA | 331 | 1.26 | TER | UAA | 98 | 0.95 | TER | UGA | 76 | 0.73 |
|  | UUG | 516 | 1.28 |  | UCG | 173 | 0.66 |  | UAG | 137 | 1.32 | Trp | UGG | 341 | 1 |
|  | CUU | 504 | 1.25 | Pro | CCU | 262 | 1.24 | His | CAU | 329 | 1.5 | Arg | CGU | 210 | 1.08 |
|  | CUC | 193 | 0.48 |  | CCC | 174 | 0.82 |  | CAC | 110 | 0.5 |  | CGC | 92 | 0.48 |
|  | CUA | 320 | 0.79 |  | CCA | 238 | 1.12 | Gln | CAA | 568 | 1.43 |  | CGA | 255 | 1.32 |
|  | CUG | 212 | 0.52 |  | CCG | 174 | 0.82 |  | CAG | 225 | 0.57 |  | CGG | 120 | 0.62 |
| Ile | AUU | 858 | 1.45 | Thr | ACU | 347 | 1.51 | Asn | AAU | 738 | 1.56 | Ser | AGU | 301 | 1.15 |
|  | AUC | 360 | 0.61 |  | ACC | 161 | 0.7 |  | AAC | 209 | 0.44 |  | AGC | 88 | 0.34 |
|  | AUA | 562 | 0.95 |  | ACA | 286 | 1.24 | Lys | AAA | 823 | 1.4 | Arg | AGA | 350 | 1.81 |
| Met | AUG | 501 | 1 |  | ACG | 127 | 0.55 |  | AAG | 351 | 0.6 |  | AGG | 135 | 0.7 |
| Val | GUU | 436 | 1.48 | Ala | GCU | 375 | 1.68 | Asp | GAU | 595 | 1.56 | Gly | GGU | 333 | 1.08 |
|  | GUC | 135 | 0.46 |  | GCC | 159 | 0.71 |  | GAC | 166 | 0.44 |  | GGC | 148 | 0.48 |
|  | GUA | 412 | 1.4 |  | GCA | 249 | 1.12 | Glu | GAA | 773 | 1.47 |  | GGA | 492 | 1.6 |
|  | GUG | 194 | 0.66 |  | GCG | 109 | 0.49 |  | GAG | 280 | 0.53 |  | GGG | 257 | 0.84 |

Table S4.7 Codons in the cp genome of *I. rubescens*

| Amino acid | Codon | No. | RSCU | Amino acid | Codon | No. | RSCU | Amino acid | Codon | No. | RSCU | Amino acid | Codon | No. | RSCU |
| --- | --- | --- | --- | --- | --- | --- | --- | --- | --- | --- | --- | --- | --- | --- | --- |
| Phe | UUU | 764 | 1.37 | Ser | UCU | 441 | 1.64 | Tyr | UAU | 618 | 1.59 | Cys | UGU | 181 | 1.35 |
|  | UUC | 351 | 0.63 |  | UCC | 249 | 0.93 |  | UAC | 157 | 0.41 |  | UGC | 87 | 0.65 |
| Leu | UUA | 681 | 1.7 |  | UCA | 330 | 1.23 | TER | UAA | 97 | 0.92 | TER | UGA | 80 | 0.76 |
|  | UUG | 511 | 1.28 |  | UCG | 182 | 0.68 |  | UAG | 138 | 1.31 | Trp | UGG | 333 | 1 |
|  | CUU | 502 | 1.26 | Pro | CCU | 261 | 1.26 | His | CAU | 331 | 1.51 | Arg | CGU | 206 | 1.05 |
|  | CUC | 185 | 0.46 |  | CCC | 170 | 0.82 |  | CAC | 107 | 0.49 |  | CGC | 93 | 0.47 |
|  | CUA | 308 | 0.77 |  | CCA | 232 | 1.12 | Gln | CAA | 567 | 1.45 |  | CGA | 263 | 1.34 |
|  | CUG | 211 | 0.53 |  | CCG | 165 | 0.8 |  | CAG | 214 | 0.55 |  | CGG | 121 | 0.62 |
| Ile | AUU | 858 | 1.45 | Thr | ACU | 351 | 1.51 | Asn | AAU | 741 | 1.56 | Ser | AGU | 312 | 1.16 |
|  | AUC | 364 | 0.62 |  | ACC | 162 | 0.7 |  | AAC | 210 | 0.44 |  | AGC | 97 | 0.36 |
|  | AUA | 550 | 0.93 |  | ACA | 287 | 1.23 | Lys | AAA | 828 | 1.4 | Arg | AGA | 363 | 1.85 |
| Met | AUG | 490 | 1 |  | ACG | 130 | 0.56 |  | AAG | 351 | 0.6 |  | AGG | 133 | 0.68 |
| Val | GUU | 429 | 1.49 | Ala | GCU | 374 | 1.65 | Asp | GAU | 597 | 1.58 | Gly | GGU | 333 | 1.08 |
|  | GUC | 130 | 0.45 |  | GCC | 160 | 0.71 |  | GAC | 158 | 0.42 |  | GGC | 151 | 0.49 |
|  | GUA | 400 | 1.39 |  | GCA | 252 | 1.12 | Glu | GAA | 766 | 1.47 |  | GGA | 490 | 1.59 |
|  | GUG | 189 | 0.66 |  | GCG | 118 | 0.52 |  | GAG | 273 | 0.53 |  | GGG | 258 | 0.84 |

| 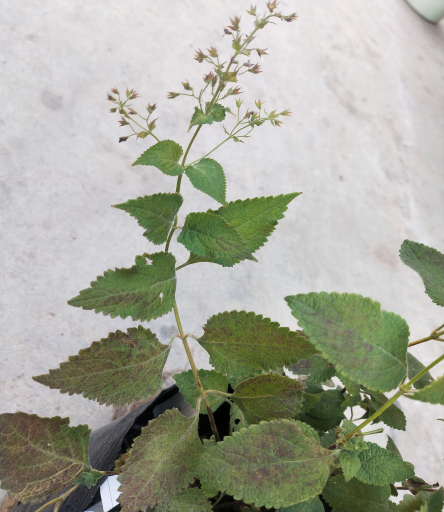 | 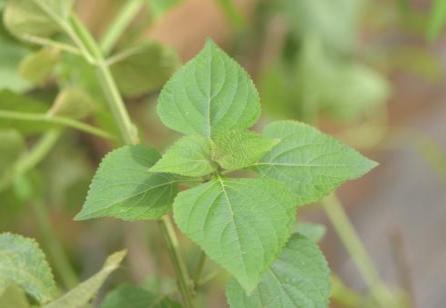 | 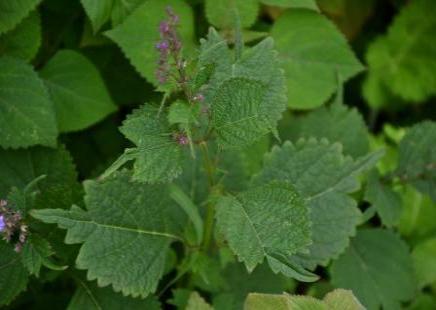 |
| --- | --- | --- |
| *I. inflexus* | *I. eriocalyx* | *I. excisus* |
| 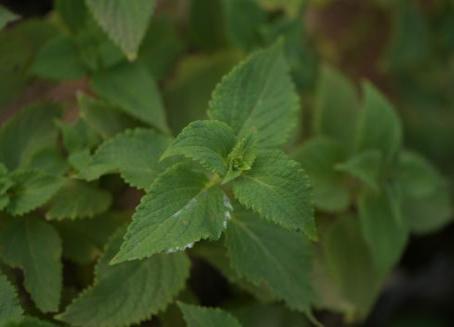 | 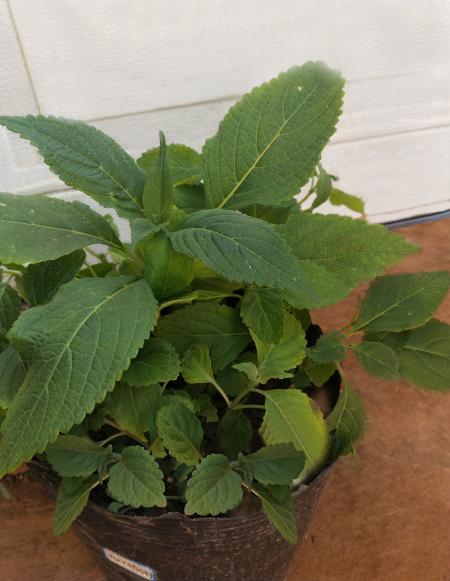 | 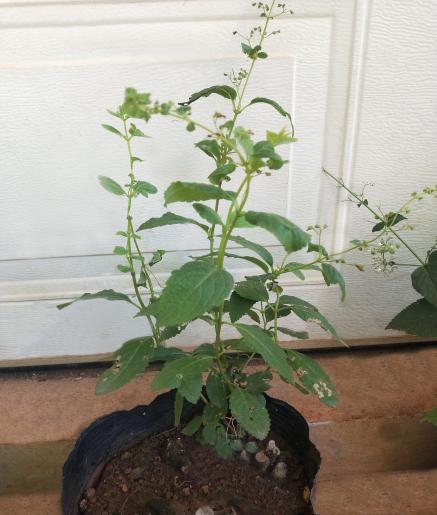 |
| *I. lophanthoides* | *I. japonicus* | *I. coetsa* |
| 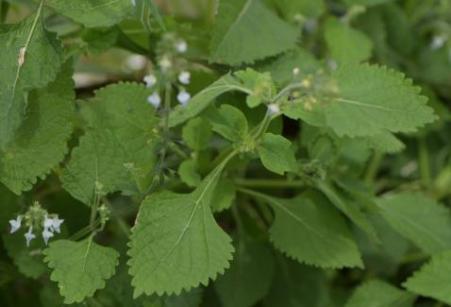 |  |  |
| *I. rubescens* |  |  |

Fig. S1 Pictures of *Isodon* plants

Fig. S2.1 Phylogenetic tree constructed using ML method based on *trnH-GUG-psbA* of cp genome

Fig. S2.2 Phylogenetic tree constructed using ML method based on *trnQ-UUG-psbK* of cp genome

Fig. S2.3 Phylogenetic tree constructed using ML method based on *trnS-GCU-trnT-CGU* of cp genome

Fig. S2.4 Phylogenetic tree constructed using ML method based on *atpH-atpI* of cp genome

Fig. S2.5 Phylogenetic tree constructed using ML method based on *trnE-UUC-trnT-GGU* of cp genome

Fig. S2.6 Phylogenetic tree constructed using ML method based on *psaA-ycf3* of cp genome

Fig. S2.7 Phylogenetic tree constructed using ML method based on *ndhC-trnM-CAU* of cp genome

Fig. S2.8 Phylogenetic tree constructed using ML method based on *psbH-petB* of cp genome

Fig. S2.9 Phylogenetic tree constructed using ML method based on *rps15-ycf1* of cp genome

Fig. S2.10 Phylogenetic tree constructed using ML method based on *rps16-trnQ-UUG* of cp genome

Fig. S2.11 Phylogenetic tree constructed using ML method based on *ccsA-ndhD* of cp genome

Fig. S3 Phylogenetic tree constructed using ML method based on combination of five IGS
